# Signaling Pathways, Chemical and Biological Modulators of Nucleotide Excision Repair: The Faithful Shield against UV Genotoxicity

**DOI:** 10.1155/2019/4654206

**Published:** 2019-08-07

**Authors:** F. Kobaisi, N. Fayyad, H. R. Rezvani, M. Fayyad-Kazan, E. Sulpice, B. Badran, H. Fayyad-Kazan, X. Gidrol, W. Rachidi

**Affiliations:** ^1^Univ. Grenoble Alpes, SYMMES/CIBEST UMR 5819 UGA-CNRS-CEA, INAC/CEA-Grenoble, Grenoble, France; ^2^Laboratory of Cancer Biology and Molecular Immunology, Faculty of Sciences I, Lebanese University, Hadath, Lebanon; ^3^Univ. Grenoble Alpes, CEA, Inserm, BIG-BGE U1038, 38000 Grenoble, France; ^4^Univ. Bordeaux, Inserm, BMGIC, U1035, F-33000 Bordeaux, France; ^5^Centre de Référence pour les Maladies Rares de la Peau, CHU de Bordeaux, France

## Abstract

The continuous exposure of the human body's cells to radiation and genotoxic stresses leads to the accumulation of DNA lesions. Fortunately, our body has several effective repair mechanisms, among which is nucleotide excision repair (NER), to counteract these lesions. NER includes both global genome repair (GG-NER) and transcription-coupled repair (TC-NER). Deficiencies in the NER pathway underlie the development of several DNA repair diseases, such as xeroderma pigmentosum (XP), Cockayne syndrome (CS), and trichothiodystrophy (TTD). Deficiencies in GG-NER and TC-NER render individuals to become prone to cancer and neurological disorders, respectively. Therefore, NER regulation is of interest in fine-tuning these risks. Distinct signaling cascades including the NFE2L2 (NRF2), AHR, PI3K/AKT1, MAPK, and CSNK2A1 pathways can modulate NER function. In addition, several chemical and biological compounds have proven success in regulating NER's activity. These modulators, particularly the positive ones, could therefore provide potential treatments for genetic DNA repair-based diseases. Negative modulators, nonetheless, can help sensitize cells to killing by genotoxic chemicals. In this review, we will summarize and discuss the major upstream signaling pathways and molecules that could modulate the NER's activity.

## 1. Introduction

The survival of living beings necessitates the preservation of genetic information encoded by DNA and its faithful transmission across generations. Hence, DNA is considered the key of life that determines the genetic makeup of a species. Maintaining this genetic stability requires an error-free process of DNA replication and the elimination, by surveying scavengers, of any DNA-damaging molecules. However, our body cells are under constant attacks by genotoxic agents such as UV and ionizing radiations, pollutants, and thermal stresses that trigger the formation of DNA lesions. Any resulting DNA damage should lead to the activation of the DNA damage response (DDR) to initiate its repair. One example of these damage/repair systems is the helix-distorting bulky adducts induced by UV radiation and repaired by a process called nucleotide excision repair (NER) [1].

Deficiencies in the NER pathway lead to various genetic disorders including the autosomal recessive disease xeroderma pigmentosum (XP), Cockayne syndrome (CS), trichothiodystrophy (TTD), cerebro-oculo-facio-skeletal syndrome (COFS), UV-sensitive syndrome (UVsS), and combined phenotypes, e.g., XP-CS and XP-TTD. These genetic disorders are characterized by premature ageing and a high prevalence of neurological disorders and cancers. Correcting of NER deficiencies is the optimal treatment for these disorders. However, considering all technological limitations for gene/cell therapy for these patients, alternative therapies such as boosting NER activities should be considered. Therefore, in this review, we will discuss the role of the major upstream pathways or molecules that modulate the NER activities and their potential uses as new therapeutic drugs helping to reduce NER disease symptoms.

## 2. Overview of the DNA Damage Response

**Figure 1:**
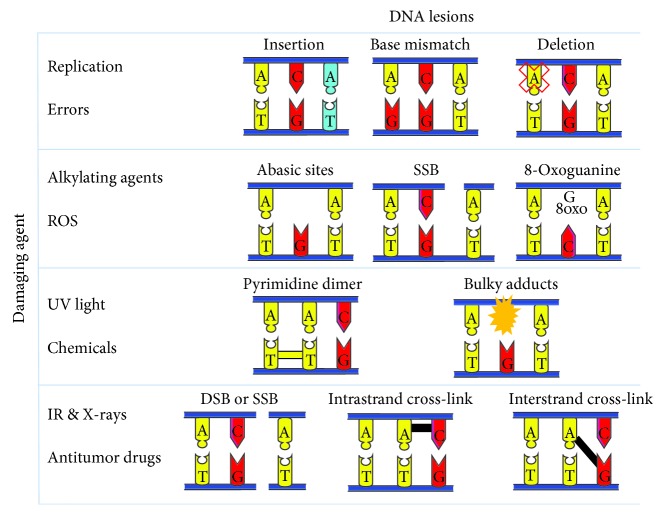
DNA lesions induced by damaging agents. Several DNA lesions are formed upon the exposure of the DNA to harmful insults. Replication errors can give rise to insertion, deletion, or base mismatch mutations. Alkylating agents and reactive oxygen species (ROS) can lead to the formation of abasic sites, single-strand breaks (SSB), and 8-oxoguanines. On the other hand, UV radiation and chemical agents mediate the formation of pyrimidine dimers and the addition of bulky adducts. Finally, ionizing radiation including X-rays and antitumor drugs can lead to single-strand breaks and double-strand breaks, together with inter- and intrastrand cross-links. ROS: reactive oxygen species; SSB: single-strand breaks; DSB: double-strand breaks; IR: ionizing radiation.

DNA damage is the outcome of a wide variety of physiological-chemical aberrations [2]. The sources of DNA damage can be either endogenous, originating from within the cell, or exogenous. The first includes reactive oxygen species (ROS) and oxygen radicals generated during cellular metabolism. These ROS can result in the formation of base modifications including abasic sites from depurination or deamination, 8-oxoguanine lesions, and single-strand breaks. It is noteworthy that ROS generation can be triggered by exogenous factors. For example, the skin contains endogenous chromophores like tryptophan, riboflavin, and mitochondrial cytochrome c oxidase that favor the generation of ROS from sunlight [3, 4]. Replication errors are other endogenous sources of DNA damage that lead to base mismatches, insertions, or deletions in the nucleotide sequence. On the other hand, external causes of DNA lesions comprise ionizing radiations, X-rays, and antitumor drugs that generate double-strand breaks (DSB), single-strand breaks, and interstrand cross-links (Figure 1) [5]. In addition, nucleotide modifications such as pyrimidine dimers and the addition of bulky adducts are triggered upon exposure to UV light or certain pollutants or chemicals (Figure 1) [4]. Therefore, a DNA damage response (DDR) is required for the correction of these DNA insults to ensure the accurate transmission of genetic information. DNA damage detection is the first step in the repair pathway, and it involves molecules termed sensors. The latter transmit signals to transducers that are mostly protein kinases [1], which in turn act on effectors, including cell cycle regulators, nucleases, and helicases, to halt the cell cycle in order to ensure DNA repair or to induce apoptosis or senescence in case of irreparable damage [6]. Any failure in this pathway can lead to the accumulation of DNA damage and ultimately premature ageing or tumorigenesis [5].

## 3. Nucleotide Excision Repair (NER)

DNA-damaging agents result in the formation of a wide variety of DNA lesions. Over the past years, several repair mechanisms specific to each lesion have been discovered. One of these mechanisms is the nucleotide excision repair (NER) required for the removal of bulky adducts and dimers. Cyclobutane pyrimidine dimers (CPD), and pyrimidine-pyrimidone photoproducts (6-4PP), are the main UV photoproducts formed following exposure to UV radiation [7]. NER starts with the recognition of the lesion and then incisions 5′ and 3′ on the damage to allow its removal creating a gap. This gap will be filled by synthesizing damage-free DNA by polymerases to finally be ligated sealing the nick [8]. NER constitutes two subpathways: global genome NER (GG-NER) that occurs all over the genome and transcription-coupled NER (TC-NER) that mends lesions in actively transcribed genes where such damages can block the progression of RNA polymerases [9]. These pathways differ mainly in the recognition step. Deficiencies in the TC-NER subpathway underlies CS and UVsS, while patients with XP, TTD, XP-CS, XP-TTD, and COFS are lacking proficiency in either GG-NER or both of the subpathways.

### 3.1. Global Genome Nucleotide Excision Repair (GG-NER)

**Figure 2:**
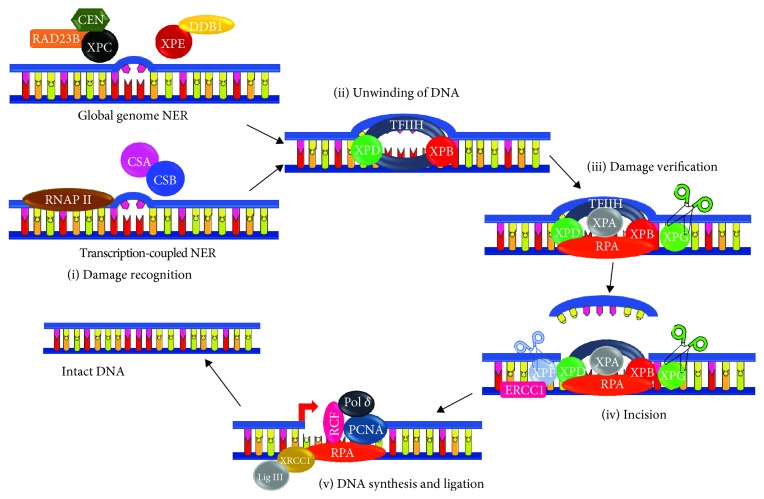
Schematic representation of nucleotide excision repair. Recognition of DNA damage differs between transcription-coupled NER (TC-NER) and global genome NER (GG-NER). (1) Damage Recognition. In TC-NER, the stalled RNA polymerase in transcriptionally active genes favors the recruitment of Cockayne syndrome proteins A and B (CSA and CSB). In GG-NER, the damage is recognized by XPC and its partners RAD23B (Rad 23 homologue B) and CEN (centrin 2) if it is a helix-distorting lesion. A mild distorting lesion in this subpathway is recognized by XPE and DDB1 (DNA damage-binding protein 1). The following steps are the same for both GG-NER and TC-NER. (2) DNA unwinding. TFIIH is recruited and it contains XPB and XPD helicases that catalyze the opening of the DNA. (3) Damage verification. XPA, XPG, and RPA1 are recruited. XPA verifies the DNA damage while RPA1 binds to the single-stranded DNA. (4) Incision. XPF that is in a complex with ERCC1 is recruited. Both XPF and XPG are nucleases that catalyze the incision of 5′ and 3′ of the damage, respectively. (5) DNA synthesis and ligation. The missing DNA sequence is synthesized by polymerase delta with the assistance of PCNA, RCF, and RPA1. DNA ligase 3 interacting with XRCC1 ligates the produced fragment leading to the formation of intact DNA. It should be noted that XPF-ERCC1 mediates the incision at the 5′ end and then the polymerase with PCNA, RCF, and PCNA will aid in displacing the damaged strand to be finally followed by the incision at the 3′ end by XPG. XPA-XPG: xeroderma pigmentosum protein A-G; NER: nucleotide excision repair; TC-NER: transcription-coupled nucleotide excision repair; GG-NER: global genome nucleotide excision repair; CSA and CSB: Cockayne syndrome proteins A and B; DDB1: DNA damage-binding protein 1; RAD23B: RAD 23 homologue B; Cen: centrin 2; TFIIH: transcription factor II H; RPA1: replication protein A; ERCC1: excision repair cross-complementation group 1; PCNA: proliferating cell nuclear antigen; RCF: replication factor C; Pol*δ*: polymerase delta; LIG3: DNA ligase 3; XRCC1: X-ray repair cross-complementing protein 1.

In GG-NER, helix-distorting lesions are initially recognized by a complex that includes XPC, Rad 23 homologue B (RAD23B), and centrin 2 (CETN2) proteins (Figure 2). RAD23B stabilizes XPC while CETN2 enhances XPC damage recognition [10]. Recent studies indicated that DNA lesions (such as CPDs) that induce very mild disruptions in the DNA double helix are poor XPC substrates. In this case, the UV-damaged DNA-binding protein (UV-DDB) complex, also known as the DDB1-DDB2 heterodimer, initially recognizes the lesions and then creates a kink that will be recognized by XPC [11]. It is noteworthy that XPC binds to the strand opposite of the adduct [8]. The DDB2-DDB1-CUL4-RBX1 E3 ligase forms a CRL4^DDB2^ ubiquitin ligase complex that ubiquitylates XPC, DDB2, and histones [12]. XPC polyubiquitination increases its affinity to DNA rather than its degradation [10]. The next step starts with the recruitment of the preincision TFIIH complex that encompasses ATPase/helicase XPD and XPB. These proteins unwind the DNA around the lesion, XPD at 5′ and XPB at 3′ of the lesion, creating a 20-30 nucleotide bubble. This complex allows the recruitment of XPA, XPG, and replication protein A (RPA1) that bind to single-stranded DNA of the bubble [11]. XPA binds at 5′ of the bubble and interacts with the preincision complex. This interaction allows the release of TFIIH component CDK-activating kinase (CDK7) to facilitate both the recruitment of XPF-ERCC1 that binds to XPA and the release of the XPC-RAD23B complex. XPF-ERCC1 endonuclease mediates the cleavage at 5′ of the lesion. Pol *ε*/*δ*/*κ*-PCNA-RFC-RPA1, the DNA replication machinery, can then synthesize the damage-free strand while displacing the damaged one and TFIIH. Afterwards, the incision at 3′ end will be catalyzed by XPG. Finally, the generated nick will be sealed by DNA ligase. It should be emphasized that replicating cells utilize polymerase epsilon (Pol *ε*) and ligase 1 while nonreplicating cells use polymerase delta/kappa (Pol *δ*/*κ*) and ligase 3*α* and X-ray repair cross-complementing protein 1 (XRCC1) [13] (Figure 2).

### 3.2. Transcription-Coupled Nucleotide Excision Repair (TC-NER)

The TC-NER pathway is triggered once RNA polymerase progression in actively transcribed genes is blocked due to the presence of bulky adducts (Figure 2). This allows the recruitment of Cockayne syndrome group B protein (CSB), also called excision repair cross-complementing protein 6 (ERCC6), which binds the polymerase and changes the DNA conformation [14]. CSB recruits CSA, EP300, and NER factors excluding XPC and UV-DDB [11]. The CSA-DDB1-CUL4-RBX1 E3 ligase form the CRL4^CSA^ ubiquitin ligase complex that ubiquitylates CSB mediating its degradation. However, UV-stimulated scaffold protein A (UVSSA) interacts with RNAPII and delivers the deubiquitinating enzyme ubiquitin-specific-processing protease 7 (USP7) that inhibits the CSA-dependent CSB degradation [12]. In addition, CSA allows the recruitment of the nucleosome remodeling factors HMGN1, XAB2, and TCEA1 [15]. The remaining steps are similar to those of GG-NER involving the recruitment of TFIIH, incision, and synthesis (Figure 2).

### 3.3. Transcriptional and Posttranslational Regulation of NER Molecular Actors

#### 3.3.1. Transcriptional Regulation

The transcription of the different NER factors is under tight balance due to the effects of both transcription factors and their repressors. The rate-limiting factor of NER is XPA that verifies the damage and leads to the recruitment of the incision proteins. The expression of XPA is controlled by the circadian clock which is higher in daytime than at night with clock circadian regulator-aryl hydrocarbon receptor nuclear translocator-like protein (Clock-Bmal1) transcriptional activator and a cryptochrome circadian regulator-period circadian regulator (CRY-PER) transcriptional repressor to enable repair in UV-exposed cells [16]. Its expression is also regulated by other transcription factors including hypoxia-inducible factor 1 (HIF1A) that upregulates XPA expression and high-mobility group protein A1 (HMGA1) that downregulates XPA expression [17, 18].

The ATP-dependent helicases XPB and XPD favor the opening of the double strand around the site of damage. Specificity protein 1 (SP1) binds to the XPB promoter and activates its expression while hepatitis B virus x (HBx) inhibits it [19, 20]. On the other hand, XPD expression is promoted by HIF1*α* and insulin while being repressed by HBx as well [21, 22]. However, long-term exposure to glucose at high concentration can mitigate the insulin-dependent increase in XPD mRNA [23].

The expression of the GG-NER sensors, XPC and XPE, is regulated in a TP53-dependent manner [24]. Overexpression is also mediated by transactivation isoform of p63 gamma (TP63) and breast cancer 1 (BRCA1) [25, 26]. Moreover, XPC expression is upregulated by SP1, sirtuin 1 (SIRT1), ARF, MC1R, and E-cadherin but downregulated by HIF1*α* and E2F4-p130. SP1's binding sequence overlaps that of HRE at the XPC promoter. This implies that a competition exists between HIF1*α* and SP1 in the binding to the XPC promoter. HIF1*α* binds to the promoter in normal condition. Upon the exposure to UV radiation, HIF1*α* is downregulated mediating the SP1-induced increase in XPC expression [21, 22] In line with the negative regulation of the NER pathway by HIF1A, it has been shown that decreased HIF1A expression in the mouse epidermis is associated with an increase in the removal rate of UVB irradiation-induced DNA damage via direct upregulation of several components of the DNA repair machinery, such as XPC and XPB. This upregulation of NER efficiency led to decreased UVB-induced carcinogenesis in HIF1A-ablated mice [27]. On the other hand, SIRT1 stimulates the expression of XPC by preventing the nuclear localization of the E2F4-p130 transcriptional repressor. The accumulation of the latter is mediated by AKT1 activation resulting from SITR1 inhibition. SIRT1 can interact with p130 and deacetylate it [28, 29]. The role of SIRT1 in enhancing XPC expression was also reported by Ming et al. where the knockdown of SIRT1 by siRNA not only decreased XPC expression but also inhibited CPD repair [30].

Finally, the expression of the incision enzymes, XPF and XPG, is upregulated by c-Fos/AP-1 [31]. In addition, CCAAT/enhancer-binding protein gamma (CEBPG) whose expression is modulated by E2F1 and YY1 favors the overexpression of XPG [32].

#### 3.3.2. Posttranslational Regulation

*(1) Ubiquitination and SUMOylation*. CRL4^DDB2^ can ubiquitinate histones, XPC and DDB2. Ubiquitination of DDB2 leads to its degradation that can be counteracted by the deubiquitinating enzyme ubiquitin-specific-processing protease 24 (USP24) [33]. DDB2 ubiquitination and degradation require the activity of the mitogen-activated protein kinase MAPK14 that mediates the phosphorylation at serine moieties [34]. On the other hand, XPC ubiquitination enhances its DNA binding ability rather than triggering its degradation [35]. XPC SUMOylation can either promote or hinder its activity depending on the targeted residues while SUMOylation of lysine 655 favors the UV-induced XPC degradation, SUMOylation of lysine residues 81, 89, and 183 of XPC stimulates NER [36].

CSA-dependent ubiquitination leads to the degradation of CSB which could be prevented by the action of the UV-sensitive syndrome A protein (UVSSA) and its recruitment of USP7 that mediates CSB deubiquitination and phosphoinositide dependence [37]. SUMOylation of CSB enhances the recruitment of CSA [38]. Moreover, XPA ubiquitination by HERC2 (E3 ubiquitin protein ligase) leads to its degradation [39]. Finally, XPF-ERCC1 is deubiquitinated by USP45 that favors its recruitment to the site of damage [40].

**Figure 3:**
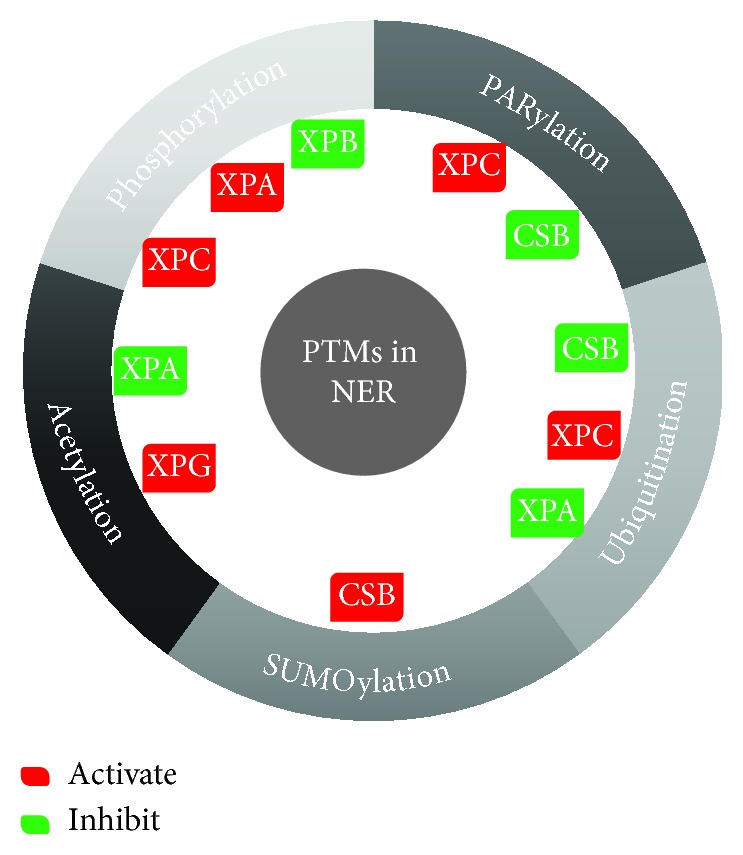
Posttranslational modifications of NER factors. Several NER factors including XPC, XPB, XPG, XPA, and CSB are subjected to different forms of posttranslational modifications that can either activate them favoring NER enhancement or the direct opposite via their inhibition.

In conclusion, the enhancement of NER can be mediated by the ubiquitination of XPC and SUMOylation of CSB and XPC but at particular residues. On the other hand, the ubiquitination of DDB2, CSB, or even XPA leads to their degradation and the impairment of NER rendering the ubiquitination enzymes targets for therapy (Figure 3).

*(2) Phosphorylation, Acetylation, and PARylation*. Phosphorylation of XPA by ataxia telangiectasia and Rad3-related protein (ATR), involved in the damage recognition of single-strand breaks, stimulates NER by blocking the HERC2-mediated ubiquitination. However, the dephosphorylation of XPA by wild-type TP53-induced phosphatase 1 (PPM1D (WIP1)) reduces NER activity [41, 42], therefore, repressing NER by inactivating XPA and XPC. The phosphorylation of XPB does not interfere with the helicase activity of TFIIH but inhibits the XPF-ERCC1 5′ end incision. XPC phosphorylation at serine 94 is mediated by casein kinase 2 (CSNK2A1) that promotes NER by allowing the recruitment of ubiquitinated XPC and other NER factors to the chromatin [43].

XPA activity is also regulated by acetylation favoring a decrease in NER activity while deacetylation enhances it by enabling the interaction with replication protein RPA132. On the other hand, the acetylation of XPG by EP300 acetyltransferase allows its accumulation at sites of damage [21].

Finally, PARylation, mediated by PARP1, regulates some of the NER proteins. First, DDB2 PARylation inhibits its ubiquitination and favors PARP1 and XPC interaction and the latter's recruitment to DNA lesions [44]. CSB is another NER protein PARylated by PARP1 leading to inhibition of its ATP hydrolysis activity [45].

Therefore, NER enhancement in this case can be mediated by phosphorylating XPA and XPC, deacetylating XPG, and PARylating XPC. NER inhibition, however, necessitates the phosphorylation of XPB, acetylation of XPA, and PARylation of CSB (Figure 3).

## 4. Modulation of the NER Pathway

**Figure 4:**
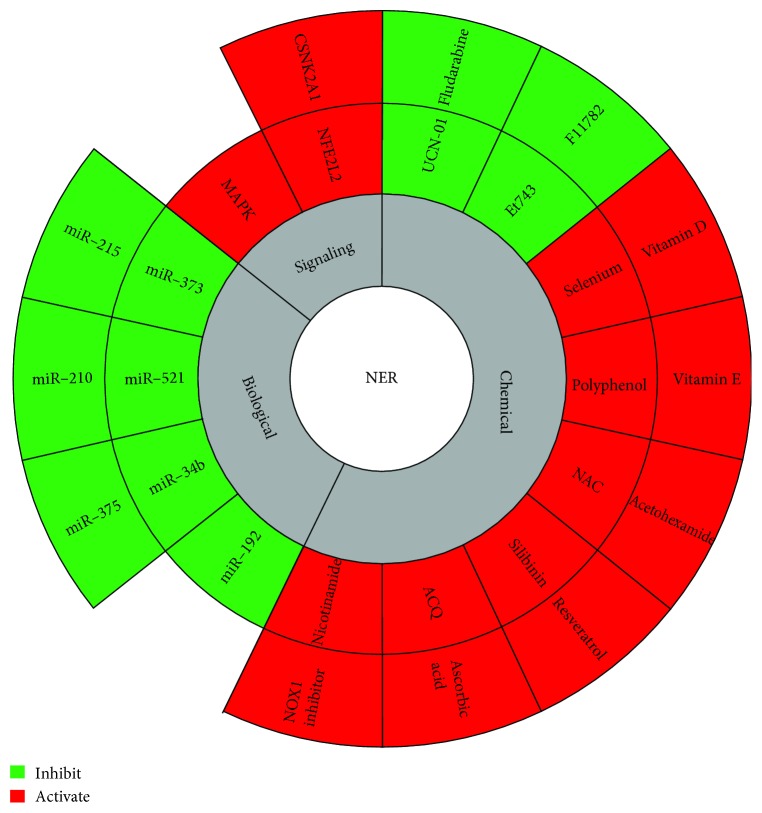
Method for the modulation of nucleotide excision repair. Nucleotide excision repair is modulated by various signaling pathways that enhance its activity including CSNK2A1, Nrf2, and MAPK. This enhancement can also be mediated by several chemical molecules. On the other hand, the inhibition of NER can be mediated by either miRNAs that target NER genes or by chemical drugs normally used in combination with chemotherapies.

NER function can be regulated by distinct signaling pathways. Moreover, this repair pathway could be modulated by the cellular redox status as it has been shown that it is inhibited by oxidative stress [46]. Moreover, molecular components such as miRNAs, being able to regulate the expression of several elements in the NER pathway [47], can affect the overall activity of this repair mechanism (Figure 4). These data suggest that NER activity can be manipulated to potentiate the repair response, thus, preventing the accumulation of DNA damage and photocarcinogenesis.

### 4.1. Signaling Pathways Regulating NER Function

#### 4.1.1. NFE2L2 (NRF2) Signaling Pathway

Oxidative stress products, including peroxidized lipids, inhibit NER [48]. *trans*-4-Hydroxy-2-nonenal (4-HNE), one of the lipid peroxidation products, inhibits NER capacity in the host cell reactivation framework [49]. On the other hand, the inflammation-derived monochloramine (NH2CL) inhibits NER via the inhibition of TP53 phosphorylation [50]. Langie et al. showed that the exposure of epithelial cells to hydrogen peroxide decreases the NER capacity to less than 50% [46]. Therefore, since NER is inhibited by oxidative stress, antioxidants can help prevent such inhibition. One of such mechanisms can be achieved by Nrf2, a transcription factor enabling the expression of antioxidant genes. NFE2L2 has a basic leucine zipper motif (bZip), allowing it to interact with other bZip-containing proteins, and another basic region that binds DNA by hydrogen bonds to favor transcription [51]. NFE2L2 activity is inhibited once bound to Kelch-like ECH-associated protein 1 (KEAP1) that triggers its ubiquitination by the ubiquitin ligase Cul 3. Binding of 4 ubiquitin residues leads to the degradation of NFE2L2 [52]. In oxidative stress conditions, the cysteine residues of KEAP1 are oxidized leading to a change in KEAP1 conformation and the ultimate release of NFE2L2 from the KEAP1-Cul 3 complex. The free NFE2L2 will be translocated to the nucleus where it forms a complex with MAF transcription factors and binds to DNA at an antioxidant-responsive element (ARE) [53]. This binding allows the transcription of antioxidant enzymes including catalase, glutathione S-transferase (GST), glutathione reductase (GR), and superoxide dismutase [54–56]. PARP1 (poly (ADP) ribose polymerase) enhances NFE2L2 transcriptional activity. It forms a complex with antioxidant response element (ARE) in NFE2L2 target genes enhancing the latter's activity without physically binding or even populating it. However, PARP1 is said to interact with MAF proteins and ARE to enhance NFE2L2 interaction with ARE. For that, PARP1 acts as a transcriptional coactivator [57]. Other coactivators include CBP/EP300 that binds and acetylates NFE2L2 at multiple lysines promoting NFE2L2-specific binding [58]. It is noteworthy that Nrf2 can be inhibited by BACH1 that competes with it for the binding to ARE. The phosphorylation of BACH1 by MAPK1 abolishes this role [59].

Notably, the NFE2L2 pathway is involved in the protection of keratinocytes, melanocytes, and fibroblasts against the harmful effects of UV radiation.

**Figure 5:**
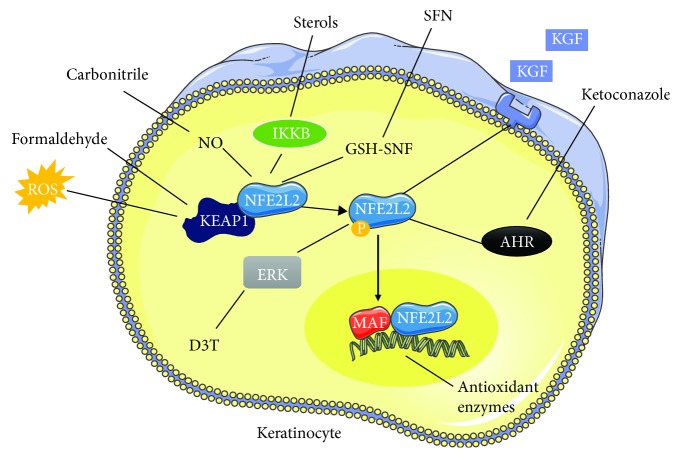
Modulation of the NFE2L2 pathway in keratinocytes. Several compounds can potentiate the NFE2L2 response in keratinocytes leading to enhanced expression of antioxidant enzymes. The binding of keratinocyte growth factor (KGF) to their receptors, activation of MAPK1 by D3T, and activation of AHR by ketoconazole all enhance NFE2L2 transcriptional activity. On the other hand, ROS, formaldehyde, carbonitriles, sterols, and sulforaphane (SFN) lead to the release of NFE2L2 from KEAP1 mediating its activity each in its unique mechanism. Activated NFE2L2 will be translocated to the nucleus to interact with MAF and favor expression of antioxidant enzymes.

*(1) Keratinocytes*. Keratinocyte growth factor (KGF) binds to its receptors to increase the proliferation of keratinocytes and elevate their NFE2L2 activity. This activity is essential against ROS generated during aerobic respiration or during the protection of the skin against pathogens [60, 61]. In addition, coal tar activates the aryl hydrocarbon receptor (AHR) that binds to the NFE2L2 gene and increases its expression [62]. Extracellular activators of NFE2L2 in keratinocytes are numerous, for instance, arsenic, a potent carcinogen that increases ROS levels enabling an increase in NFE2L2 activity [63]. In addition, xenobiotics like formaldehyde, eugenol, or dinitrochlorobenzene bind covalently to cysteine of KEAP1 enabling NFE2L2 release and activation [64]. This release is also mediated by either plant sterols that activate IKB while IKK*β* will bind to KEAP1 preventing its interaction with NFE2L2 [65] or by carbonitriles that nitrosylate KEAP1 cysteine [66]. Other activators include flavonoids that protect cells from UV radiation [67]. Sulforaphane (SFN) found in broccoli and Brussels sprouts reduces GSH levels altering KEAP1 conformation releasing NFE2L2 and enhancing expression of antioxidant enzymes [68]. Furthermore, D3T increases the mRNA levels and elevates phosphorylation of NFE2L2 by MAPK1 kinase [69]. Finally, ketoconazole activates AHR to increase NFE2L2 transcriptional activity [70] (Figure 5).

However, it should be noted that NFE2L2 overactivation leads to abnormal proliferation of keratinocytes inducing hyperkeratosis due to the expression of various downstream target genes. Among those genes is *EPIGEN*, encoding a growth factor, which causes the enlargement of the sebaceous gland and cyst formation all via EGFR signaling [71]. Small proline-rich protein 2d (*SPRR2D*), another NFE2L2 target gene, weakens the epidermal barrier leading to inflammation and enhances keratinocyte proliferation. Finally, secretory leukocyte peptidase inhibitor (SLPI) mediates hyperkeratosis [72].

**Figure 6:**
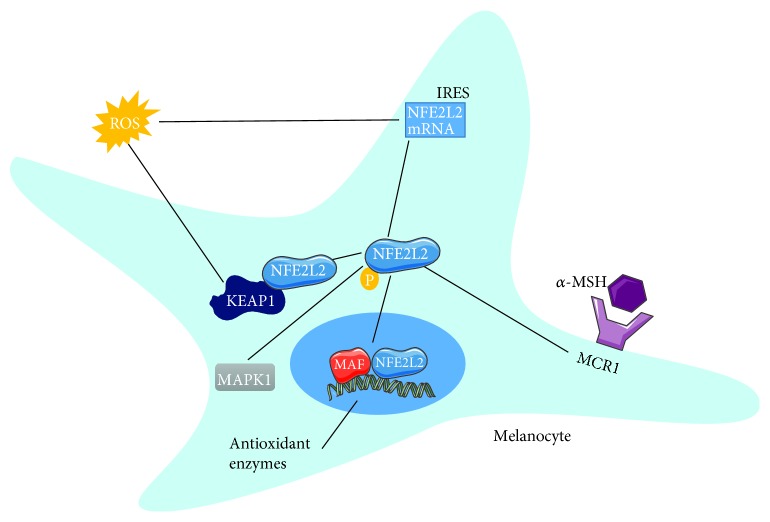
Modulation of the NFE2L2 pathway in melanocytes. The expression of antioxidant enzymes in melanocytes is mediated by the NFE2L2 pathway whose activity can be enhanced due to the effects of ROS that mediated the release of NFE2L2 from its inhibitor KEAP1 or through the enhancement of its mRNA expression. Moreover, activated MAPK1 or even *α*-MSH binding to its receptor MCR1 increases NFE2L2 activity where it is translocated to the nucleus to interact with MAF and express the enzymes. It should be noted that the activation of NFE2L2 necessitates its phosphorylation mainly by MAPK1.

*(2) Melanocytes*. Melanocytes produce melanin in a process termed melanogenesis which requires tyrosinases possessing both a diphenylene activity leading to H_2_O_2_ generation and a catalase activity for H_2_O_2_ decomposition. NFE2L2 is required to protect these cells against the generated ROS during melanin synthesis [73]. NFE2L2 activation can be mediated by the binding of melanotropin (*α*MSH) to MC1R to form a complex that initiates the transcription of ARE-containing genes [74]. Stress that leads to an unbalanced redox status sends a signal to IRES (internal ribosome entry sites) in NFE2L2 mRNA increasing its synthesis [75]. In addition, ERK1/2 activation by the RAS/RAF/Mek/MAPK1 pathway favors the phosphorylation of NFE2L2 [76]. Extracellular activators of NFE2L2 include afamelanotide and curcumin even though the latter increases apoptosis of keratinocytes [54, 77] (Figure 6).

**Figure 7:**
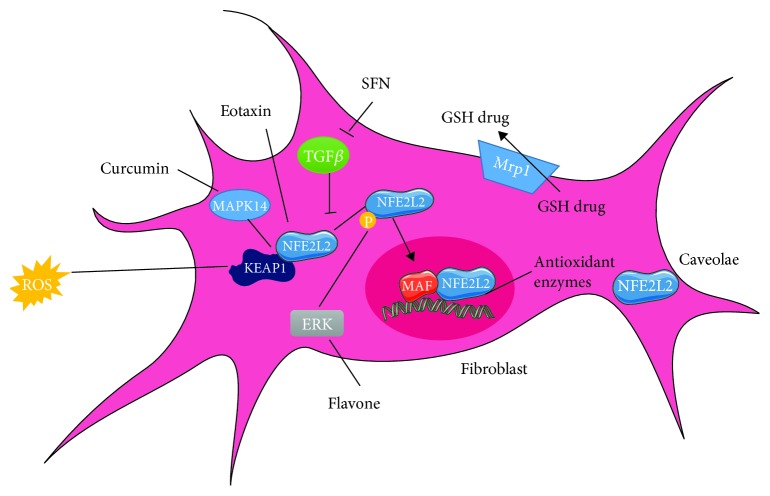
Modulation of the NFE2L2 pathway in fibroblasts. The NFE2L2 pathway in fibroblasts can be either activated or inhibited depending on the administered compound. ROS, eotaxin, and curcumin mediate the release of NFE2L2 from the inhibitor KEAP1. Flavone via the activation of MAPK1 facilitates the phosphorylation of NFE2L2. In addition, Mrp1 removes glutathione conjugates to drugs in a NFE2L2-dependent manner. However, TGF*β* signaling inhibits NFE2L2 activity which can be counteracted by the administration of SFN. Finally, the fibroblast caveolae can also inhibit NFE2L2 function preventing the expression of antioxidant enzymes that require the translocation of a NFE2L2 transcription factor to the nucleus and its interaction with MAF.

*(3) Fibroblasts*. Hydrogen peroxide induces NFE2L2 activation as well as expression of antioxidants and antiapoptotic proteins [78]. Does eotaxin chemokine also mediate an increase in NFE2L2 expression or activation together with flavone that activates MAPK1 mediating NFE2L2's phosphorylation [79, 80]? Moreover, curcumin disrupts TGF*β* signaling by the phosphorylation of SMAD2 and increases the TGF-induced factor, a TGF*β* inhibitor, leading to NFE2L2 activation and the ultimate decrease of ROS [81]. On the other hand, thioredoxin inhibits NFE2L2 due to its free thiol that prevents KEAP1 oxidation [82]. The caveolae formed by a dent fibroblast membrane can favor NFE2L2 degradation. Finally, diethyl malate increases the expression of Mrp1 that removes glutathione conjugates with harmful substances including drugs only in NFE2L2-positive cells [83] (Figure 7).

NRF1was also linked to the enhancement of NER. Keratinocytes with Nrf1 loss are sensitive to killing by UVB exposure. Analysis of DNA damage repair in the cell population surviving UVB exposure showed a decrease in CPD repair by slot blot assay. Han et al. linked the latter to a decrease in XPC expression as it was reversed by the overexpression of XPC in Nrf1-inhibited cells enabling the repair. It is not through the inhibition of XPC repressors that Nrf1 mediated such function but rather due to the maintenance of GSH levels [84]. Therefore, the stimulation of the NRF pathway by these various compounds should be further explored as a method for NER enhancement.

#### 4.1.2. AHR Pathway

The aryl hydrocarbon receptor (AHR) is found in the cytosol and it senses various chemicals including flavonoids and dioxin. The binding of ligands to the AHR induces a conformational change favoring its translocation to the nucleus. There, it will bind to the xenobiotic response element (XRE) of genes to increase their transcription. One of these target genes is cytochrome P450 family enzyme CYP1A1. The latter allows the detoxification of pollutants and generates ROS and mutagenic metabolites [85]. This receptor was shown to decrease the clearance of CPD in the course of global genome repair. AHR-compromised cells manifested elevated CPD repair in a CDKN1B-dependent manner as the latter was increased in AHR-silenced cells and enhanced NER [86].

However, other studies have shown that some ligands of AHR including ketoconazole and quercetin do not lead to ROS generation but induce an antioxidant response due to the activation of NFE2L2-NQO1 [87].

Cynaropicrin (Cyn), a sesquiterpene lactone found in artichoke, is an antioxidant that activates the AHR-NFE2L2-NQO1 pathway. Cyn favors the activation and translocation of AHR to the nucleus which in turn mediates the nuclear translocation of NFE2L2 and the increase in NFE2L2 and NQO1 (NAD (P) H-quinone oxidoreductase 1) mRNA levels. In addition, it decreased the levels of ROS and proinflammatory cytokines IL6 and tumor necrosis factor *α* (TNF*α*) [88]. Other phytochemicals that operate on the AHR-NFE2L2-NQO1 pathway include the *Labisia pumila* that decreases the UVB-induced TNF*α* production [89] which is also reduced by curcumin that reduces IL6 production as well [90]. Finally, phenols from *Lonicera caerulea* and *Vaccinium myrtillus* fruit protect keratinocytes against UVB-induced ROS generation and IL6 production [91].

#### 4.1.3. PI3K/AKT1 Pathway

The PI3K/AKT1 pathway is induced following tyrosine kinase receptor-mediated activation of PI3K. The latter converts PIP2 (phosphatidylinositol biphosphate) into PIP3 allowing the recruitment of both PDK1 (phosphoinositide-dependent kinase) and AKT11 allowing the phosphorylation of its threonine 308. In addition, the target of rapamycin complex 2, MTORC2, phosphorylates serine 473 of AKT1. All that leads to AKT1 activation that can be counteracted by PTEN phosphatase [92]. The involvement of this pathway in the modulation of NER function is still controversial. AKT activates MDM2 that favors degradation of TP53 [93]. Therefore, AKT1 inhibits NER due to the decrease in XPC and DDB2-TP53-dependent transcription [94]. Another mode of inhibition is mediated by the AKT1-dependent localization of XPC transcriptional repressors (P130). This is reversed by the action of deacetylase and longevity factor (SIRT1) that deacetylates PTEN to inhibit AKT1. In addition, the abundance of lysine acetylation sites in GG-NER proteins DDB1, DDB2, CUL4A, and RAD23A suggests that they can be possibly regulated by deacetylases including SIRT1 [29]. Moreover, AKT1 is known for its contribution in cell cycle progression as it prevents the translocation of CDKN1A and CDKN1B cell cycle inhibitors, stabilizes cyclin, and increases metabolic activity by the phosphorylation and inhibition of GSK [95–97] Further, it activates nuclear factor kappa B (NFK*β*), IAPS, CASP8, and FADD-like apoptosis regulator (FLIP) but inactivates the proapoptotic protein Bad and caspase 9. Nonetheless, AMP-activated protein kinase (AMPK) can counteract the AKT1-mediated MTOR activation during UV radiation [92]. Finally, AKT1 can enhance TC-NER via the phosphorylation of EP300 that relaxes the chromatin to allow the recruitment of repair factors [98].

#### 4.1.4. MAPK Pathway

MAPK pathway starts with RAS-GTP that allows the stimulation of MAPK-KK (RAF) activating MAPK-K (MEK) via phosphorylation that further on actuates MAPK (ERK1/2, JNK, or MAPK14) by dual phosphorylation of tyrosine and threonine residues. MAPK1 controls differentiation and proliferation while MAPK14 and JNK regulate apoptosis, cell cycle arrest, invasion, and others [99]. It has been reported that ERK1/2 activation enhances NER and, hence, decreases mutagenicity [100]. In addition, this MAPK posttranslationally modifies RAD23A, RAD23B, and RPA12 [101]. Similarly to AKT1, MAPK can also mediate phosphorylation of EP300, where upon UV radiation, EP300 is recruited to the damage site in heterochromatin and is phosphorylated by both MAPK14 MAPK and AKT1 increasing histone acetyl transferase (HAT) activity to acetylate H3 and H4 leading to chromatin relaxation. After that, EP300 phosphorylation conditions it for proteasomal degradation enabling the recruitment of DDB2, XPC, and other NER factors [102]. However, the MAPK pathway can also mediate metastasis. High ROS levels or UVA radiation activates MAPKs JNK, MAPK1, and MAPK14 leading to activation of AP-1, c-Jun and c-FOS mediating the expression of matrix metalloproteinase 1 (MMP1) [103]. MMPs break down extracellular matrixes and are highly expressed by XPC fibroblasts with enhanced MMP1 promoter activity and ROS activation leading to the predisposition to invasive skin carcinomas [104]. Moreover, Zhao et al. demonstrated a role of MAPK14 in mediating the repair of CPD by utilizing SB203580, MAPK14 inhibitor, and measuring the repair kinetics using slot blot assay. They also reported a MAPK14-dependent ubiquitination of DDB2 favoring its degradation and clearance form damaged chromatin [34].

#### 4.1.5. CSNK2A1 (CK21) Pathway

Casein kinase 2 (CSNK2A1) is a serine/threonine kinase involved in various signaling pathways including PI3K/AKT1, epidermal growth factor receptor (EGFR), heat shock protein 90 (Hsp90), and nuclear factor kappa B (NFK*β*) [105–110]. It was recently linked to the regulation of single- and double-strand break repair [111, 112]. One of the CSNK2A1 substrates is X-ray repair cross-complementing protein 1 (XRCC1) involved in the ligation step of both nucleotide and base excision repairs. CSNK2A1-mediated phosphorylation of XRCC1 enhances the stability of the XRCC1-ligase III complex [112, 113]. The involvement of CSNK2A1 in DNA repair pathways rendered it an important target for combinatorial therapies with adduct-inducing agents like cisplatin. The damage induced by the latter, if not repaired by NER, gives rise to double-stranded breaks at stalled replication forks and the accumulation of *γ* H2AX. In a study by Drygin et al., the treatment with CX-4945, CSNK2A1 inhibitor, induced formation of tails in the course of alkaline comet assay and the accumulation of phosphorylated *γ* H2AX. This thus confirms the role of CSNK2A1 in mediating repair of single-strand breaks during the late stages of nucleotide excision repair [114]. In another study by Im and Nho on idiopathic pulmonary fibrosis fibroblasts, they discovered a reduction in expression of PUMA and caspase-3/7 following cisplatin treatment thus signifying a decrease in DNA damage-induced apoptosis. They also noted an increase in XRCC1 activity as a result of CSNK2A1 hyperactivation causing a drop in *γ* H2AX levels [115]. CSNK2A1 is also required for the phosphorylation of XPC at serine 94, as mentioned previously promoting the CPD and 6-4 PP repair [43].

### 4.2. Chemical Compounds

Much effort is focused, nowadays, on identifying components that can modulate NER activity. During the last years, several distinct chemical compounds have been characterized for their ability to modulate NER activity in either a positive or negative manner.

#### 4.2.1. Nicotinamide

Nicotinamide (vitamin B3) is a precursor for NAD (nicotinamide adenine dinucleotide), a coenzyme for ATP production. The supplementation of nicotinamide can reverse the ATP depletion that occurs during chromatin remodeling and DNA repair [116]. This chemical does not prevent the formation of CPD or 8-oxo-guanine, but it enhances their removal. It favors the increase of unscheduled DNA synthesis after UV radiation and reduces UV-induced immunosuppression [117]. In addition, it prevents UV-induced glycolytic blockade and restores the ATP and NAD levels [118, 119]. Nicotinamide can have a potential role in the chemoprevention of arsenic-induced skin cancer. Arsenic is a UV radiation cocarcinogen found in contaminated water and it mediates DNA damage. The administration of nicotinamide prior to arsenic treatment combined with UV irradiation decreases the levels of CPDs and 8-oxo-guanines [120]. Nonetheless, nicotinamide supplementation also rescues the mitochondrial phenotype in XPA cells. The latter are characterized with reduced mitochondrial autophagy and increased membrane potential speculated to be the cause of neurodegeneration in XPA patients. The treatment of XPA cells with either nicotinamide riboside or nicotinamide mononucleotide promotes the NAD^+^-SIRT1-PGC-1*α* axis attenuating PARP effect and reversing the mitochondrial dysfunction [121].

#### 4.2.2. NOX1 Inhibitor

NADPH oxidase (NOX) allows the generation of ROS following UVB radiation. Several NOX isoforms (NOX1 to NOX5, DUOX1, and DUOX2) exist. These proteins differ in their tissue distribution, and they are involved in regulating different biological processes such as cellular signaling, differentiation, and regulation of gene expression. NOX proteins' activation requires their assembly with others which in case of NOX1 are NOXA1, NOXO1, p22^phox^, and small Rac GTPase [122]. NOX1 activation in XPC keratinocytes via the DNA-PK/AKT1 axis elicits their neoplastic transformation [123]. Therefore, the inhibition of NOX1 might have a positive effect on NER. This blockade can be mediated by the utilization of peptide inhibitors that target either the proline-rich region of NOXO1 or that of the SH3 domain of NOXA1 necessary for NOX1 function. Their utilization enhances CPD and (6-4) PP repair and decreases apoptosis by reducing activation of caspases 3, 8, and 9 [124]. Moreover, the same study revealed that CDK4, CDK6, cyclin (B, D1, and E), and CDC25C were increased following NOX inhibition while CDKN2A and CDKN1A were reduced enabling cell cycle progression. On the other hand, NOX inhibition can also be mediated by syringic acid that blocks the NOX/PTP-*κ*/EGFR axis [125].

#### 4.2.3. Silibinin

It is a bioactive flavonolignan present in milk thistle. It has been reported that upon human dermal fibroblast exposure to UVB radiation, the treatment with silibinin increased TP53 and GADD45*α* expression and reduced the formation of CPDs. The same study also showed that silibinin increases the protein levels of XPA, in a TP53-dependent manner, together with XPG and XPF. At the mRNA level, only XPA but neither XPG nor XPF levels was increased, indicating that the increase in their protein levels can be mediated by silibinin's regulation at translational levels rather than its role in the enhancement of transcription [126]. Furthermore, silymarin, whose major active ingredient is silibinin, has been shown to inhibit photocarcinogenesis via the inhibition of UV-induced ROS, inflammation, and immunosuppression. These flavonolignans accelerate the repair of CPDs and increase the expression of XPC and XPA despite the lack of a UVB radiation filtering effect [127].

#### 4.2.4. NAC

N-Acetylcysteine (NAC) is a nontoxic analogue of cysteine with antioxidant activity that detoxifies mutagens. It can be deacetylated to produce cysteine, a precursor of GSH. NAC decreases the formation of DNA adducts and its intracellular metabolites are scavengers of ROS [128]. The administration of NAC to UVB-irradiated normal human epidermal keratinocytes can partially inhibit the production of UVB-induced cytokine IL6 and TNF*α* [88]. NAC has also been demonstrated to reduce the formation of 8-oxoguanines and lipid peroxidation in rats treated with sodium fluoride. This suggested a possible role of NAC in antagonizing the damage induced by sodium fluoride [129]. In addition, the combination of NAC treatment with acetyl-L-carnitine prior to irradiation causes an increase in DNA damage-related factor including XPC [130].

#### 4.2.5. ACQ

ACQ is an alanine cysteine glutamine tripeptide with antioxidant activity compared to that of glutathione. Its activity was tested in keratinocytes and fibroblasts treated with hydrogen peroxide. The administration of ACQ decreased the positive staining with H_2_DCFA signifying a decrease in the levels of reactive oxygen species. Hence, it can protect cells against H_2_O_2_ treatment [131]. This capacity was proven to reduce NER activity. However, the use of ACQ to enhance NER activity has not been established so far. Therefore, it would be of great importance to check this correlation in future studies [46].

#### 4.2.6. Ascorbic Acid

Ascorbic acid (ASA) is an antioxidant that protects DNA from damage [132]. It has also an anti-inflammatory potential as it suppresses NFK*β* [133]. It has been established that in keratinocytes subjected to UVA radiation, the pretreatment with ASA prevents the changes in ROS, GSH, and lipid peroxidation levels; thus, it counteracts the UVA-induced oxidative stress. Since the change of GSH levels can modulate the expression level of XPC and lipid peroxidation hinders NER, the use of ASA for NER enhancement should be further explored [48, 84]. Moreover, it has also been reported that ascorbic acid decreases the phosphorylation of MAPK14 and MAPKAPK2 inhibiting UVA-induced MAPK activation together with the reduction of apoptosis through a decrease in caspase 3 activations [134].

#### 4.2.7. Resveratrol

Resveratrol (RSV) has a para-hydroxyl group conveying a scavenging activity of free radicals [135]. As proven by various *in vitro* studies, it can prevent H_2_O_2_- and UV-induced oxidative damage [136]. RSV activates the NFE2L2 pathway and induces the transcription and translation of glutamyl-cysteinyl ligase and glutathione peroxidase 2 (GPX2) and increases GSH levels. This NFE2L2 activation and increase in GSH levels render this molecule of interest for NER enhancement even though such role has not been explored yet. RSV does not induce the synthesis of NFE2L2 but it enhances its stability and nuclear accumulation. The NFE2L2 activation is mediated by the MAPK pathway mainly by MAPK1 [3].

#### 4.2.8. Selenium

Selenium is a micronutrient that can be potentially used in cancer prevention. This potential role was examined in a study done on men with elevated risk of prostrate cancer in New Zealand in 2004. Those individuals were provided with either a placebo or selenium supplementation in the form of selenised yeast for 6 months, and DNA damage was assessed by comet assay. The results show an inverse correlation between DNA damage and selenium status [137]. A different study also reported a role of selenium in the repair of CPD adducts. A chloramphenicol acyl transferase reporter in a plasmid was inactivated by CPDs and then transfected into fibroblasts supplemented with selenium. The reporter activity was restored signifying the repair of the induced CPD adducts [138]. Selenium is also involved in enhancing the repair of oxidative DNA damage like 8-oxoguanine. These lesions are mainly repaired by 8-oxoguanine glycosylase (OGG1) where the oxidation of its redox-sensitive residues leads to the attenuation of OGG1's activity. Selenium induces antioxidant selenoproteins that help maintain OGG1 in a reduced active form. The effects of selenium can be due to (i) selenium metabolites that are not associated with proteins or (ii) selenoproteins with selenium in the form of selenocysteine [139].

#### 4.2.9. Polyphenols

Polyphenols can be found in a variety of plants including green tea leaves and grape seeds. Nowadays, various studies are focusing on the use of polyphenols in skin cancer prevention due to their potential role in photocarcinogenesis inhibition. Green tea polyphenols (GTP) were found to prevent UV-induced immunosuppression while also decreasing the amount of CPDs. The latter effect was speculated to be the result of GTPs' enhancement of XPA, XPC, and RPA1's mRNA expression [140]. Green tea polyphenols are also effective in the reduction of inflammation markers (COX2, PGH2, PCNA, and cyclin D) and proinflammatory cytokines (TNF*α*, IL6, and IL1*β*) [141]. GSP (grape seed proanthocyanidins) mediates similar effects as GTP concerning the repair of CPD as well as enhanced XPA, XPC, RPA11, and DDB2 expression. In addition, it favors the nuclear translocation of XPA and augments its interaction with ERCC1 [142].

#### 4.2.10. Vitamin E

The exposure of the skin to UV radiation causes depletion of vitamin E that quenches ROS [143]. One study revealed that the treatment with the antioxidant *α*-tocopherol, vitamin E, leads to both direct and indirect protections. The first is mediated by the quenching of free radicals while the latter is via the increased epidermal thickness [144]. Krol et al. also revealed that the topical application of alpha tocopherol on mouse skin inhibits the formation of cyclobutane pyrimidine dimers. This suggests a role of vitamin E in the protection against UV-induced skin photodamage. However, the applied vitamin E is rapidly depleted in a dose-dependent manner following UV radiation [145].

#### 4.2.11. Acetohexamide

This compound is an antidiabetic drug targeting ATP-sensitive potassium channels, and it belongs to the family of sulfonylureas regulating insulin secretions. However, a new function was attributed to acetohexamide following a chemical screen performed on BRCA1-mutated cells. These cells are deficient in base excision repair (BER) and hence accumulate oxidative stress. The screen consisted of treating the cells with a chemical library then transfecting them with GFP plasmid containing oxidized bases. Acetohexamide was then identified as a chemical molecule that enabled the repair of the oxidized bases in BER-deficient cells and that was signified by the increase in GFP expression [146]. Another chemical screen-based study also proved the importance of such compound in enhancing DNA repair but of pyrimidine dimers rather than oxidized bases. They proved that acetohexamide enhances the removal of pyrimidine dimers in cells deficient in both the global and transcription-coupled nucleotide excision repairs. The actual mechanism is still not clear but it involves the downregulation of MUTYH protein as knockout of this BER protein gave similar results compared to the drug treatment [147].

#### 4.2.12. Vitamin D

The active form of vitamin D is 1,25-hydroxy-cholecalciferol that can either be provided through diet or through the conversion of 7-dehydrocholesterol in the skin mediated by the exposure to UV light. It was reported that keratinocytes with intact vitamin D receptor (VDR) show accelerated repair of CPDs compared to counterparts with knocked out VDR [148]. Treatment of cells with 1,25 dihydroxyvitamin D3 increases the expression of XPC and DDB2 [149].

#### 4.2.13. Ecteinascidin 743 (Et743)

Also known as Trabectedin or Yondelis is an FDA-approved antitumor chemotherapy drug. It binds to and alkylates guanine residues at the N^2^ position in the minor groove bending the DNA towards the major groove opposite to the adduct. The mechanism of inhibition occurs at the TC-NER. Treatment of TC-NER-proficient cells with Et743 generates more single-strand breaks compared to cells with mutations in TC-NER-involved proteins. The proposed mechanism based on bacterial homologs is that this drug traps endonucleases after the cleavage step preventing the ligation of the DNA [150].

#### 4.2.14. F11782

The reported mechanism of action of F11782 is the inhibition of topoisomerases I and II. It is a 2′,3′-bis-0pentafluorophenoxyacetyl-4′,6′-ethylidene-*β*-D-glucoside of 4′-phosphate-4′-dimethylpipodophylloyoxin 2-N-methyl glucamine salt. However, in a DNA damage detection (3D) assay, Barret et al. reported the role of F11782 in the inhibition of NER using UVC-damaged plasmid incubated with cell extract and biotinylated dNTPs. F11782 was found to inhibit the incision step of NER rather than the repair synthesis stage. Despite that, the actual target of drug mediating such inhibition is still unknown [151].

#### 4.2.15. Fludarabine

Fludarabine or Fludara is a purine nucleoside analog used as chemotherapeutic medication for the treatment of leukemia and lymphoma [152, 153]. The effect of this drug on NER was reported by Yamauchi et al. using an alkaline comet assay to measure the amount of DNA strand breaks at the incision step and monitoring the rejoining of the DNA after repair via the incorporation of tritiated thymidine. The pretreatment with Fludarabine inhibited thymidine incorporation and allowed the preservation of the comet tail up to 4 hours post UV with no sign of repair [154].

#### 4.2.16. UCN-01

UCN-01 is known to block cell cycle checkpoints and inhibit protein kinase C [155]. Jiang and Yang tested the effects of this drug on cisplatin-induced DNA damage normally repaired by NER. UCN-01 was found to inhibit repair as tested by repair synthesis assay and host cell reactivation. This is probably mediated by the hindrance of XPA-ERCC1 interaction despite the fact that no direct interaction between the drug and NER proteins was reported. They speculated that UCN-01's effect might be through the regulation of signaling pathways enabling posttranslational modifications of NER proteins [156].

### 4.3. Biological Molecules

MicroRNAs (miRNAs) are small noncoding RNA molecules with a seed region in their 5′ end that interacts with the 3′UTR of mRNA leading to the latter's degradation or block of translation. They can also interact with 5′UTR and coding regions. miRNAs are involved in regulating a broad range of biological processes including DNA damage and repair genes [157]. For instance, miR-192 whose expression is induced by HBV infection can bind and inhibit mRNA of ERCC3 and ERCC4 [47]. Moreover, hypoxia can induce the downregulation of RAD23B via the upregulation of miR-373. The utilization of anti-miR-373 was able to reverse the hypoxia-induced Rad23B reduction [158]. CSA which is part of the TC-NER recognition was also proven to be suppressed by miR-521 [159]. In addition, a study conducted by Tessitore et al. utilizing irradiated NSCLC A549 cells showed the upregulation of miR-34b that targets ERCC5 in addition to miR-192 and miR-215 that both target ERCC3, ERCC4, and XPA [157]. Furthermore, Crosby et al. showed that forced overexpression of miR-210 leads to the suppression of RAD52 [47]. On the other hand, Friboulet et al. revealed that miR-375 was reduced in ERCC1-positive cancers and its predicted targets are PARP4, ERCC3, TP53, and USP1 [160] (Table 1). Since a wide range of miRNAs can control the expression of DNA repair genes, summarized in Table 1, it would be of great interest to analyze the miRNA profile of NER-deficient cells. This analysis will enable the pinpointing of miRNAs with possible roles in the persistence of lesions by downregulating repair enzymes paving a road for the utilization of anti-miR to counteract their effects.

## 5. Conclusion

Bulky adducts induced by UV radiation can be removed by nucleotide excision repair (NER). In this review, we focused on factors that could modulate NER activity. These factors include signaling pathways, chemical compounds, and interfering RNAs. The use of chemical compounds shows great promise as many of them are dietary supplements with limited toxicity and have the abilities to modify several signaling pathways. As for RNA interference, the issue of effective and specific delivery is still under study and provides a hindrance for their use clinically. On the other hand, gene therapy for XP is being investigated which could provide promise in replacing the mutated NER genes rather than increasing NER activity but this technique is still highly experimental. Regulation of XP proteins by epigenetics can also enhance NER for that it should be further investigated. The modulation of NER can help shield UV rays and possibly eliminate their genotoxic effects on the formation of cancer.

## Figures and Tables

**Table 1:** miRNAs that regulate NER genes.

miRNA	Target mRNA	Target mRNA alternative name
miR-373	RAD23B	

miR-521	CSA	ERCC8

miR-34b	XPG	ERCC5

miR-192	XPB	ERCC3
	XPF	ERCC4
	XPA	

miR-215	XPB	ERCC3
	XPF	ERCC4
	XPA	

miR-210	RAD52	

miR-375	PARP4	
	XPB	ERCC3
	TP53	
	USP1	
